# Joint Image Reconstruction and Super-Resolution for Accelerated Magnetic Resonance Imaging

**DOI:** 10.3390/bioengineering10091107

**Published:** 2023-09-21

**Authors:** Wei Xu, Sen Jia, Zhuo-Xu Cui, Qingyong Zhu, Xin Liu, Dong Liang, Jing Cheng

**Affiliations:** 1 Shenzhen Institutes of Advanced Technology, Chinese Academy of Sciences, Shenzhen 518055, China; wei.xu@siat.ac.cn (W.X.); sen.jia@siat.ac.cn (S.J.); zx.cui@siat.ac.cn (Z.-X.C.); qy.zhu@siat.ac.cn (Q.Z.); xin.liu@siat.ac.cn (X.L.); 2 University of Chinese Academy of Sciences, Beijing 101408, China

**Keywords:** MR imaging, multi-task, image reconstruction, super-resolution, deep learning

## Abstract

Magnetic resonance (MR) image reconstruction and super-resolution are two prominent techniques to restore high-quality images from undersampled or low-resolution k-space data to accelerate MR imaging. Combining undersampled and low-resolution acquisition can further improve the acceleration factor. Existing methods often treat the techniques of image reconstruction and super-resolution separately or combine them sequentially for image recovery, which can result in error propagation and suboptimal results. In this work, we propose a novel framework for joint image reconstruction and super-resolution, aiming to efficiently image recovery and enable fast imaging. Specifically, we designed a framework with a reconstruction module and a super-resolution module to formulate multi-task learning. The reconstruction module utilizes a model-based optimization approach, ensuring data fidelity with the acquired k-space data. Moreover, a deep spatial feature transform is employed to enhance the information transition between the two modules, facilitating better integration of image reconstruction and super-resolution. Experimental evaluations on two datasets demonstrate that our proposed method can provide superior performance both quantitatively and qualitatively.

## 1. Introduction

Magnetic resonance imaging (MRI) has been widely used in modern medical diagnosis as a non-invasive imaging modality. The raw data acquired in MRI are located in k-space; however, the imaging time for MR examination is relatively long due to the data acquisition scheme in k-space and the time for signal recovery. Prolonged scan time not only leads to patient discomfort but also affects the image quality, particularly in high-resolution (HR) imaging that demands even longer scan time. High-resolution images, due to their rich anatomical information and textural details, have the potential to significantly enhance the accuracy of medical diagnosis. Therefore, reducing imaging time and improving image quality are of paramount importance in MR imaging.

Acquiring less data in k-space is one of the most important ways to accelerate MR acquisition, and high-quality images can be obtained through image restoration from the partial k-space data. Generally, there are two mainstream approaches for image restoration: image reconstruction (Rec) and super-resolution (SR). The former aims to recover artifact-free images from undersampled k-space data, while the latter infers high-resolution (HR) images from low-resolution (LR) observations. Undersampling in k-space preserves imaging resolution in the stage of data acquisition; however, it introduces artifacts in the image due to the violation of the Nyquist sampling theorem. In the case of SR, although data are sampled with the Nyquist sampling ratio, the absence of data fidelity in the high frequency of k-space is prone to generating fake structures, creating puzzles in the images. Furthermore, image reconstruction usually takes place in k-space domain [1,2] or hybrid domain [3,4], while super-resolution is performed in image domain. To improve image quality, prior information is employed in both tasks, such as sparse priors [3,4,5]. Although optimization-based techniques have achieved numerous successes in MR Rec and SR, there are still some limitations in practice, such as long restoration time, heavy computational burdens, and challenges in hyper-parameter selection, which mainly include sparse transformations, regularization parameters, and the number of iterations. Moreover, the use of simplistic priors restricts the performance of MR restoration in achieving better image quality.

Recently, deep learning (DL)-based methods have made significant advancements and demonstrated remarkable improvements in various fields [6,7,8,9,10]. Specifically, DL has gained popularity in image restoration tasks and has shown great potential in enhancing image quality. In MR reconstruction, neural networks are employed to eliminate undersampled artifacts in the image domain or utilized as regularization in an iterative algorithm with end-to-end training [11,12,13,14,15,16,17,18,19,20,21,22,23,24,25,26,27]. For SR, neural networks learn the mapping between LR and HR image pairs [28,29,30,31,32,33,34,35,36,37]. Research in SR focuses on designing advanced network architectures to enhance the structures of interest and preserve sensitive detail information in the generated HR images. However, most studies generate LR images by degrading in the image domain, overlooking the intrinsic properties of MR imaging, where raw data are acquired in k-space using multi-channel coils.

Although the implementation details are different, both Rec and SR tasks aim to obtain high-quality images with fine details from partially acquired k-space data. The Rec task ensures data consistency, while the SR task focuses on generating perceptual structures and improving the spatial resolution of the image. By leveraging the strengths of both tasks, it is possible to accelerate MR imaging and achieve higher image quality. Currently, there are two common approaches for integrating Rec and SR: performing the tasks separately or combining them sequentially using two separate sub-networks for Rec and SR. However, these approaches have limitations. Performing the tasks separately can lead to information loss and error propagation, while sequential approaches overlook the correlations between the two tasks. In this work, we propose a novel multi-task framework for joint MR Rec and SR. Instead of the sequential approaches of combining Rec and SR, we introduce a transmission of features from the Rec task to the SR task. This feature transmission facilitates the integration of information from both tasks, allowing the SR task to benefit from the enhanced features obtained during the Rec task. By jointly considering Rec and SR tasks and incorporating feature transmission, we aim to improve the overall performance of MR image restoration, resulting in higher image quality and faster MR imaging.

The main contributions of this work are as follows:

- We introduce a novel multi-task framework that integrates the model-based deep learning reconstruction (Rec) method with the super-resolution (SR) method, resulting in a reduction of the scan time for MR imaging and an enhancement in image quality.
- The proposed method performs image reconstruction and super-resolution simultaneously, demonstrating superior performance compared to various sequential combinations of state-of-the-art MR Rec and SR methods, as well as other multi-task approaches.
- We validate the efficiency of the proposed method on different datasets, including a 2D brain dataset and a 3D T1w brain and neck vessel wall dataset.

## 2. Related Works

### 2.1. DL-Based MR Image Reconstruction

DL-based MR reconstruction methods aim to remove artifacts caused by undersampling. In 2016, Wang et al. [11] applied CNN to MR reconstruction, setting a precedent for DL-based reconstruction. Subsequent studies focused on advanced networks to learn the mapping between the zero-filled image and the fully sampled one, and combining the imaging model with deep networks. For instance, Zbontar J et al. [12] proposed a Unet-based network, and Lee et al. [13] proposed a ResNet-based network for MR image reconstruction. The main drawback of the data-driven approach is its lack of interpretability, as it does not consider the problem from the perspective of the MR imaging model. Model-driven methods then gained lots of attention in MR image reconstruction [14,15,16,17,18,19,20,21,22,23,24,25,26,27]. Model-based reconstruction algorithms have inspired network innovations in model-driven methods to make them more flexible and interpretable [38]. For example, ISTA-Net [15] and its variants [17] unroll the Iterative Shrinkage Threshold Algorithm (ISTA) to solve the problem of MR image reconstruction. Aggarwal et al. [16] proposed integrating a CNN-based denoiser into model-based reconstruction. ADMM-CSNet [18] unrolls the alternating direction multiplier method (ADMM) [39] for compressed sensing applications. Advanced networks, such as generative adversarial networks (GAN) [40] and attention mechanisms, are also employed in MR image reconstruction [14,24,25,26,27].

### 2.2. DL-Based MR Image Super-Resolution

With the success of deep learning in the field of image classification and recognition, deep learning has also found applications in image SR. The first DL attempt for image SR was the SRCNN [28], and since then, numerous DL-based SR methods have been proposed. For instance, Oktay et al. [41] introduced a residual convolutional neural network (CNN) to recover the resolution of cardiac images. This network handles multiple input data from different observation perspectives, thereby enhancing its performance. To address the challenge of partial loss of image details, Shi et al. [42] extended the SRCNN framework and designed a network that combined multi-scale global residual learning and local residual learning based on shallow network blocks. Local residual learning is utilized to recover high-frequency details of images. The lack of high-quality medical image training samples has been a concern, leading to many SR models suffering from over-fitting or under-fitting. To mitigate this issue, Zhao et al. [43] proposed the channel splitting network (CSN) to alleviate the representation burden of deep models. GAN has also been applied in the field of SR to recover texture details of SR images [29,30,31,32,33,34]. Building on the concept of GAN, MedSRGAN [44] was introduced for the SR of medical images, including MR images, allowing for the generation of more realistic texture details. Additionally, the architecture of transformer [7] has been applied to image SR [35,36,37]. Transformer can effectively capture remote and non-local features that facilitate image SR. In conclusion, deep learning has significantly advanced the field of image SR, and the incorporation of various advanced network architectures has contributed to more practical and efficient solutions in image super-resolution.

## 3. Methods

### 3.1. Problem Statement

The multi-coil super-resolution MR forward imaging model can be formed as:

(1) b=PFCx+δ

where *b* is the acquired undersampled, low-resolution k-space data, *x* is the MR image to be recovered, *P* is the sampling mask that P=MH, *M* is the binary undersampling mask, *H* is the downsampling operator to crop out the high-frequency components of the k-space domain, *F* is the Fourier transform, and *C* is the coil sensitivity map; δ denotes the measurement error which can be well modeled as noise. Our objective is to reconstruct an artifact-free SR image *x* from the given partial k-space data *b*.

However, Equation (1) is an ill-posed problem making it challenging to directly obtain the image through operator inversion. With the aid of deep learning, we propose a network *G* with parameter θ to learn the best estimate of the inversion process. The network *G* takes undersampled, low-resolution MR image $x^{LR}$ as input and generates artifact-free SR image *x* and the corresponding fully sampled high-resolution image $x^{GT}$ as ground truth, and the loss function *L* is used to measure the error between *x* and $x^{GT}$. Therefore, the training process of the network can be formulated in the following supervised manner:

(2) $$\theta_{G}^{*}=\underset{\theta_{G}}{\arg\min}\;E_{x}\left[L\left(G\left(x^{LR};b;M;C;\theta_{G}\right),x^{GT}\right)\right]$$

### 3.2. Overall Architecture

**Figure 1:**
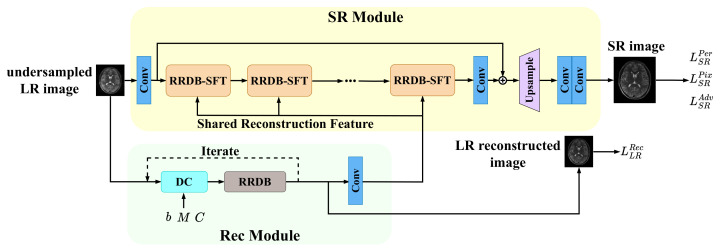
The overall architecture of our proposed network. RRDB: Residual in Residual Dense Block, SFT: Spatial Feature Transform, DC: Data Consistency.

Our proposed network, shown in Figure 1, can simultaneously perform image super-resolution and MR image reconstruction. The network takes an undersampled, low-resolution MR image as input, along with the input coil sensitivity map, undersampling mask, and undersampled k-space, and outputs super-resolution MR images corresponding to fully sampled, high-resolution images as ground truth. The network comprises two main components: an SR Module and a Rec Module. The SR Module extracts features from the undersampled and low-resolution MR image and restores the resolution of the image. The Rec Module performs the MR image reconstruction process in LR space. Since the SR Module depends on the output of the Rec Module, we first introduce the Rec Module, followed by the SR Module, and finally the loss function used to optimize the network.

#### 3.2.1. Rec Module

For the Rec Module, the reconstruction problem can be modeled as solving the following minimization problem:

(3) $$x_{rec}^{*}=\underset{x_{rec}}{\arg\min}\;\frac{1}{2}||Ax_{rec}-b{\left|\right|}_{2}^{2}+R\left(x_{rec}\right)$$

$x_{rec}$ is the LR image to be reconstructed, $R(x_{rec})$ is the regularization term, *b* is the k-space data, and A=MFC is the coding matrix in MRI.

Following the strategy of unrolling, we select the proximal gradient descent (PGD) algorithm [45] to solve the problem (3), resulting the following iterations:

(4) $$\begin{array}{cc} x_{rec}^{k+1} & =prox_{R,\eta }(x_{rec}^{k}-\eta \nabla \frac{1}{2}||Ax_{rec}-{b||}_{2}^{2})\\ \; & =prox_{R,\eta }(x_{rec}^{k}-\eta A^{H}\left(Ax_{rec}-b\right) \end{array}$$

where η is a step size and the proximal operator prox with respect to the function *R* is defined as follows:

(5) $$prox_{R,\tau }\left(p\right)=\underset{p}{\arg\min}\left\{R\left(t\right)+\frac{||t-{p||}_{2}^{2}}{2\tau })\right\}$$

The proximal operator $prox_{R,\tau }\left(\cdot \right)$ is a nonlinear operator that can be replaced by a trainable network Γ, so the final iterative process is as follows:

(6) $$\left\{\begin{array}{cc} s^{k} & =x_{rec}^{k}-\eta A^{H}\left(Ax_{rec}^{k}-b\right)\\ x_{rec}^{k+1} & =\Gamma (s^{k}) \end{array}\right.$$

**Figure 2:**
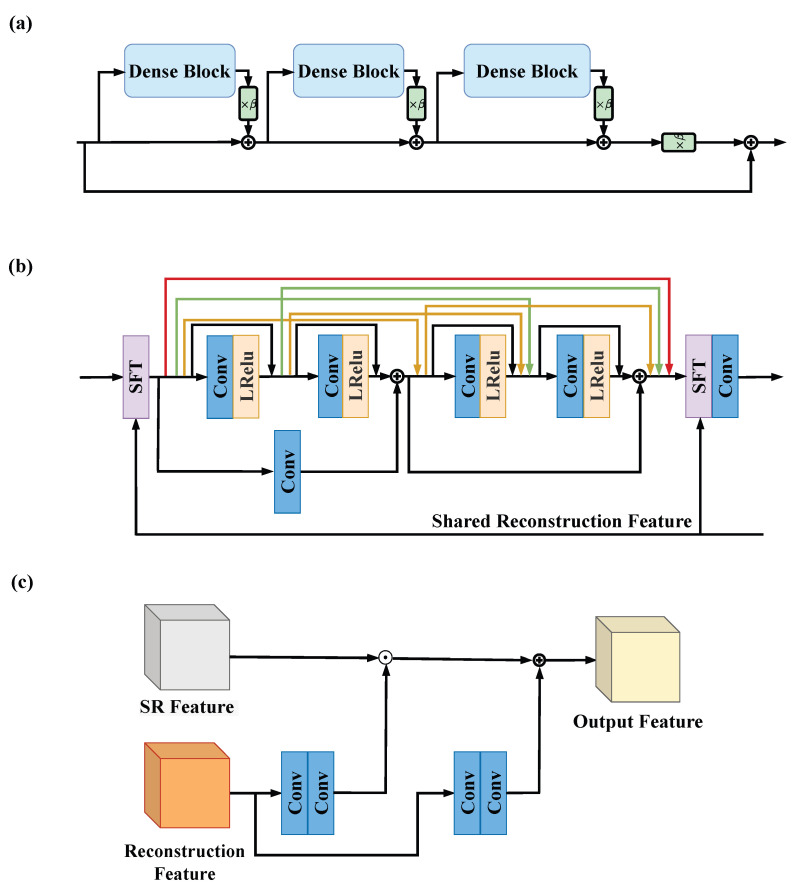
(**a**) The architecture of Residual in Residual Dense Block (RRDB). β=0.2 is a scaling parameter. (**b**) The architecture of Dense Block in RRDB. (**c**) The detailed structure of Spatial Feature Transform (SFT) layer.

We make step size η a trainable parameter and then the iterative process in (6) is refined into a trainable network, called PGD Net. In PGD Net, the first step involves data consistency, which enforces data fidelity. For the proximal operation, we employ the Residual in Residual Dense Block (RRDB) [31], as shown in Figure 2a. To enhance the stability of the Reconstruction Module, we utilize the deep equilibrium model [46] with Jacobian-Free back-propagation [47] to ensure the convergence of iterations and reduce the training memory.

The PGD Net will generate LR reconstructed images after completing the iteration process. The reconstructed LR image is represented as a reconstruction feature by a convolutional layer, and finally transmitted to the SR module.

#### 3.2.2. SR Module

The SR Module comprises two parts: feature extraction and upsampling recombination. In the feature extraction part, an initial convolutional layer is utilized to represent the input image as a high-dimensional feature map, facilitating subsequent deep feature extraction. Following the convolutional layer, 23 SR blocks are connected. These blocks can be any feature extraction blocks, and in this work, we use the architecture of RRDB. The RRDB benefits from the combination of residual network [48] and dense connections [49], enhancing its ability to extract complex features and patterns in images, thereby improving the model’s super-resolution capability. In addition, we add two skip connections in each Dense Block of RRDB to enhance the mobility of the features. To enable the SR Module to receive the reconstruction features from the Rec Module, we configure the Spatial Feature Transform (SFT) layer [30] within the RRDB, thus constituting the RRDB-SFT (shown in Figure 2). The SFT layer performs an affine transformation on the intermediate features, with the transformation parameters obtained by transforming two convolutional layers under an additional prior condition. Here, the prior condition is the reconstruction feature from the Rec Module. To improve efficiency, the reconstruction feature is broadcasted to all SFT layers, meaning that all SFT layers share the same reconstruction feature. This approach better guides the SR Module with the assistance of the Rec Module, resulting in a higher-quality image recovery process.

In the upsampling recombination part, there is an upsampling operation on the low-resolution feature map obtained earlier, and the upsampling method used here is nearest neighbor interpolation. The following two convolutional layers are used to suppress the artifacts caused by the upsampling operation and recombine the feature map to generate the final SR image.

#### 3.2.3. Loss Function

To reduce the average pixel difference between recovered images *x* and Ground Truth $x^{GT}$, pixel loss is introduced:

(7) $$L_{SR}^{Pix}=E_{x}||G\left(x^{LR}\right)-x^{GT}|{|}_{1}$$

Perceptual loss [50] can enhance the semantic correlation between recovered images *x* and Ground Truth $x^{GT}$, and it contributes to the generation of more realistic texture details [29], which is defined as follows:

(8) $$L_{SR}^{Per}=E_{x}||\phi \left(G\left(x^{LR}\right)\right)-\phi \left(x^{GT}\right)|{|}_{1}$$

where ϕ is a pre-trained VGG-19 network [51].

To enhance the contribution of MR image reconstruction, reconstruction loss $L_{LR}^{Rec}$ is introduced:

(9) $$L_{LR}^{Rec}=E_{x_{Rec}}\left|\right|x_{rec}-x_{LR}^{GT}|{|}_{1}$$

where $x_{rec}$ is the output of PGD Net; $x_{LR}^{GT}$ is the image obtained by inverse Fourier transform of the LR k-space without undersampling.

GAN can facilitate the network to generate realistic texture details close to the Ground Truth image [29]. We use the network *G* as the generator and VGG-19 network [51] as the discriminator *D*, and let them perform adversarial training to improve the realism of generated SR images. Therefore, the adversarial loss of generator $L_{SR}^{Adv}$ and the adversarial loss of discriminator $L_{SR}^{Dis}$ are introduced:

(10) $$L_{SR}^{Adv}=-E_{x}\left[\log D\left(G\left(x^{LR}\right)\right)\right]$$

(11) $$L_{SR}^{Dis}=-E_{x}\left[\log\left(1-D\left(x\right)\right)\right]-E_{x^{GT}}\left[\log D\left(x^{GT}\right)\right]$$

Therefore, the overall loss function of the generator *G* is as follows:

(12) $$L^{G}=L_{SR}^{Per}+\gamma_{Pix}L_{SR}^{Pix}+\gamma_{Rec}L_{LR}^{Rec}+\gamma_{Adv}L_{SR}^{Adv}$$

where $\gamma_{Pix}$, $\gamma_{Rec}$, $\gamma_{Adv}$ are trade-off parameters of different losses.

### 3.3. Datasets

Two sets of data were used to evaluate the feasibility of the proposed method. One is a set of 2D multi-contrast brain data; these contrasts include T1w, T2w, T1 FLAIR and T2 FLAIR. The other is a set of 3D T1w brain and neck vessel wall imaging (VWI) data. The two datasets were acquired with Cartesian, rectilinear k-space sampling. Informed consent was obtained from the imaging subjects in compliance with the Institutional Review Board (IRB) policy.

The fully sampled HR k-space data were acquired, and we simulated to generate LR undersampled data by cropping and undersampling. The LR-HR paired images were generated from the corresponding LR-HR k-space data through coil combination with ESPIRiT map. This process allowed us to obtain LR images and their corresponding HR counterparts for training and evaluation purposes.

**Figure 3:**
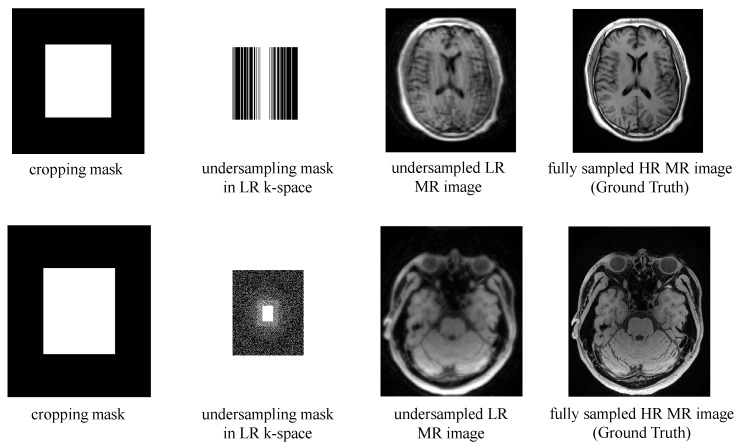
The cropping masks, undersampling masks in LR k-space and examples of undersampled LR MR image and fully sampled HR MR image sample from the brain dataset (upper) and the VWI dataset (lower).

The two datasets are acquired on a 3.0T MR imaging system (uMR790, United Imaging Healthcare, Shanghai, China). The brain dataset contains 1900 fully sampled k-space slices acquired from 12 subjects. To reduce the computational complexity, we used the coil compression technique GCC [52] to compress the coils of the original data into 12. Each k-space slice has dimensions of 12 × 256 × 232 (coils × FE × PE). We used a 1D 2.5x Gaussian undersampling mask and 2x SR scaling factor to generate the undersampled LR brain data. Since cropping in the FE direction cannot accelerate MRI, the final imaging acceleration is 2 × 2.5 = 5x, and 1600 slices were used for training, 150 for validation, and 150 for testing. The 3D VWI data contains 1472 fully sampled k-space slices, which were acquired from four healthy volunteers using T1-weighted MATRIX sequences with non-selective excitation. The dimension of the k-space slices of the VWI data is 18 × 336 × 280 (coils × PE × SPE). A 2D 4x Poisson undersampling mask and 2x SR scaling were used to generate LR data, resulting in a 2 × 2 × 4 = 16x acceleration. In the VWI dataset, there are 1104, 184, and 184 slices for the training set, validation set, and test set, respectively. The cropping masks, undersampling masks used to generate the datasets, and the examples of LR-HR image pairs for both datasets are presented in Figure 3.

### 3.4. Evaluation Metrics

For a fair quantitative comparison, we use Peak Signal-to-Noise Ratio (PSNR), Structure Similarity Index Measure (SSIM) [53], and Learned Perceptual Image Patch Similarity (LPIPS) [54] to evaluate the quality of the generated images.

The PSNR is calculated based on the mean square error (MSE) between the generated image *x* and the Ground Truth image *y*. The larger PSNR indicates that the distortion of the generated image is smaller, and the specific calculation formula is as follows:

(13) $$MSE=\frac{1}{MN}\sum_{i=1}^{M}\sum_{j=1}^{N}{\left[x\left(i,j\right)-y\left(i,j\right)\right]}^{2}$$

(14) $$PSNR=10\log_{10}\left(\frac{MAX^{2}}{MSE}\right)$$

where *M* and *N* denote the size of the image, and MAX is the maximum possible pixel value in the image.

The SSIM measures the similarity of the generated image *x* and the Ground Truth image *y* based on brightness, contrast and structure, and a larger SSIM indicates better quality of the generated image, as calculated by the following formula:

(15) $$SSIM=\frac{\left(2\mu_{x}\mu_{y}+C_{1}\right)\left(2\sigma_{xy}+C_{2}\right)}{\left(\mu_{x}^{2}+\mu_{y}^{2}+C_{1}\right)\left(\sigma_{x}^{2}+\sigma_{y}^{2}+C_{2}\right)}$$

where μ and σ represent the mean and variance of the image. $\sigma_{xy}$ represents the mutual covariance of the two images x and y, and $C_{1}={\left(0.01MAX\right)}^{2}$ and $C_{2}={\left(0.03MAX\right)}^{2}$ are two constants.

The LPIPS is calculated based on the L2 distance of the generated image *x* and the Ground Truth image *y* in the feature space of a pre-trained network. This metric is more in accordance with human perception than the above two metrics. The specific calculation formula is as follows:

(16) $$LPIPS=\sum_{l}\frac{1}{H_{l}W_{l}}\sum_{i=1}^{H_{l}}\sum_{j=1}^{W_{l}}{∥w_{l}⊙\left({\hat{x}}^{l}\left(i,j\right)-{\hat{y}}^{l}\left(i,j\right)\right)∥}_{2}^{2}$$

where $w_{l}$ is a channel-wise vector, ${\hat{x}}^{l}$ and ${\hat{y}}^{l}$ are the output feature map of the *l*-th layer of a pre-trained network, by convention we use AlexNet [6] and $H_{l}$ and $W_{l}$ are the size of the feature maps.

### 3.5. Training Details

In our work, we used the ADAM optimizer [55] with default parameters $\beta_{1}=0.9$, $\beta_{2}=0.999$ and $\epsilon =1\times 10^{-8}$ to optimize the parameters of the generator and discriminator. Both the generator and discriminator were trained with a learning rate of $1\times 10^{-4}$, and the learning rates were reduced by half after 50k, 100k, 200k, and 300k iterations. The batch size for training is 4, and the sizes of the input undersampled LR MR images for the brain dataset and VWI dataset are 128×116 and 168×140, respectively. As for the trade-off parameters of different losses, to ensure the perceptual quality of the generated super-resolution images and to highlight the role of MR image reconstruction, we set $\gamma_{Rec}$ to 1, $\gamma_{Pix}$ to $1\times 10^{-2}$, and $\gamma_{Adv}$ to $5\times 10^{-3}$. All the experiments are implemented by the PyTorch framework on two NVIDIA Tesla V100 GPUs.

## 4. Experimental Results and Analysis

### 4.1. Ablation Study

In this subsection, we present the results of ablation experiments conducted to validate our motivation that integrating Rec into SR can generate images with higher quality. The quantitative results on the brain and VWI datasets are presented in Table 1 and Table 2, respectively. In these two tables, the following notations are used: “SR” and “Recon” represent using only the SR or Rec Module to obtain fully sampled HR images; “Recon-SR” refers to training reconstruction and SR separately; “Recon+SR” denotes the sequential combination of reconstruction and SR with end-to-end training. The main difference between the proposed method and “Recon+SR” is that in our approach, the Rec Module and SR Module are not simply connected in series, but they are interactively connected using SFT layers. This interactive connection allows for more effective information exchange between the two modules, resulting in improved image quality.

Comparing the results of the SR scheme and the Recon scheme, it can be observed that the PSNR and SSIM of the SR scheme are relatively lower, while the LPIPS is significantly higher than that of the Recon scheme, indicating that the MR images generated by the SR scheme are closer to the original images in terms of visual perception but hold a higher level of distortion.

In our attempt to combine super-resolution with MR image reconstruction, we developed the Recon-SR scheme. However, this scheme performs poorly in terms of PSNR and SSIM compared to the Recon scheme. In particular, it can be seen that there is a substantial decrease in SSIM in the brain dataset, indicating that the Recon-SR scheme did not result in accurate MR images. The reason for this result is that super-resolution and MR image reconstruction are still two independent processes, and the results of reconstruction in the LR space produce errors that are amplified by the SR network. These errors cannot be reduced by optimizing the SR network, and at the same time, the SR network also produces errors, resulting in the accumulation of errors. As a result, the Recon-SR scheme was not effective in improving the accuracy of MR image reconstruction and super-resolution.

The Recon+SR scheme performs better than the Recon-SR scheme in all metrics. This is because the errors generated by the Rec network can be optimized with end-to-end training, alleviating the problem of error accumulation that occurs when the two processes are performed independently. Comparing our proposed method with other schemes, our proposed method performs the best in all metrics, demonstrating that the MR images generated by our network have precise structural information and texture details, making the visual perception close to the original image. These results suggest that the interactive connectivity scheme employed by our network is superior to the scheme that simply connects the Rec module and the SR module in series and that the scheme that combines MR image reconstruction with super-resolution is superior to the scheme that uses either technique alone.

**Figure 4:**
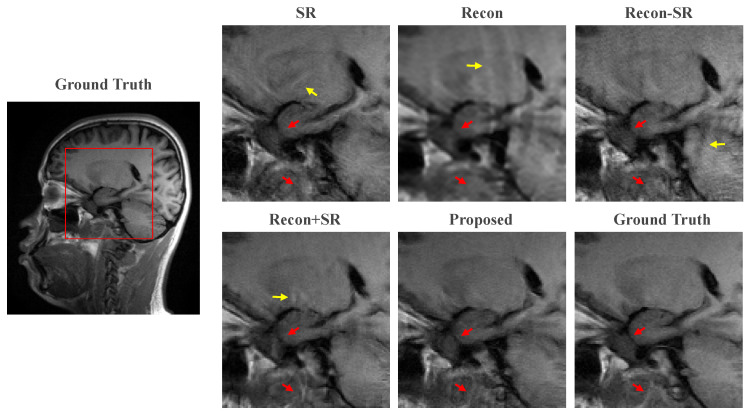
Zoomed-in view of the ablation experiment. The yellow arrows point to the fake structures in the images and the red arrows represent the fine details that can be recovered by our proposed method compared to other methods.

The qualitative comparisons are shown in Figure 4. It can be seen from the zoomed-in images that the proposed method can effectively preserve fine details and exhibits the best consistency with the ground truth. In the case of the only SR task, the restoration tends to produce fake structures, as indicated by the yellow arrow. Furthermore, visible artifacts are present in the image generated by the Recon method. Comparing Recon+SR with Recon-SR, it can be observed that the end-to-end training in Recon+SR yields better results, indicating that the joint training approach benefits image restoration more than separate training. The proposed method demonstrates its capability to enhance image quality and maintain the fidelity of fine details, outperforming the other approaches in the qualitative evaluation.

### 4.2. Comparison Experiment

To comprehensively evaluate the performance of our proposed model, we conducted a series of comparison experiments on the two datasets. Specifically, we compared our method with ten other models, which include nine Recon+SR methods and one state-of-the-art multi-task method called T2Net [56]. For the nine Recon+SR methods, we selected three reconstruction algorithms (CG-SENSE [57], MoDL [16], and DL-ESPIRiT [20]) and three super-resolution algorithms (bicubic, MedSRGAN [44], and BebyGAN [34]). These algorithms were combined sequentially to form the nine comparison methods. To ensure a fair comparison, each of these methods was carefully optimized to guarantee the best performance and ensure equitable evaluations.

The quantitative results of different methods on the two datasets are presented in Table 3 and Table 4. These results were obtained on the entire testing dataset. Across both datasets, our proposed method outperforms all other competing methods. The highest PSNR values indicate that our method can generate images with the least distortion, while the highest SSIM values suggest that it retains more global and local structural information. Moreover, the lowest LPIPS scores indicate that our method achieves the closest visual perception to the ground truth.

**Figure 5:**
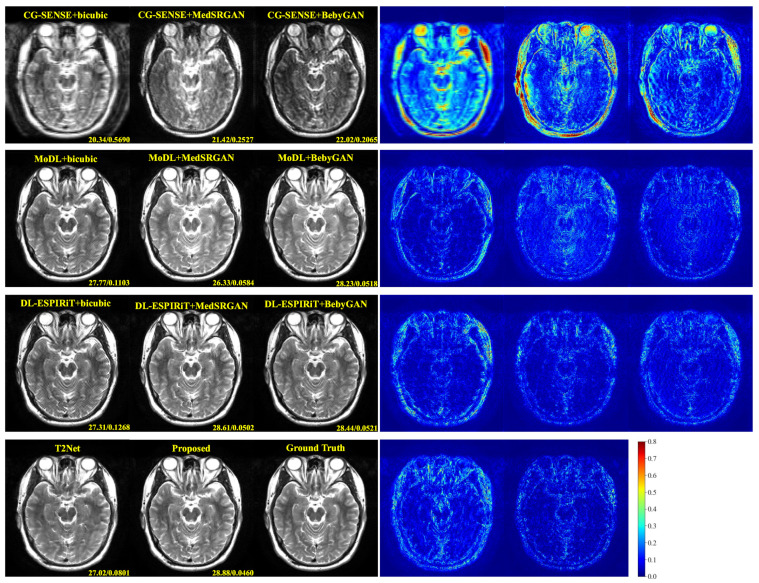
Visual comparison of each method for a slice of axial view in the brain dataset.

**Figure 6:**
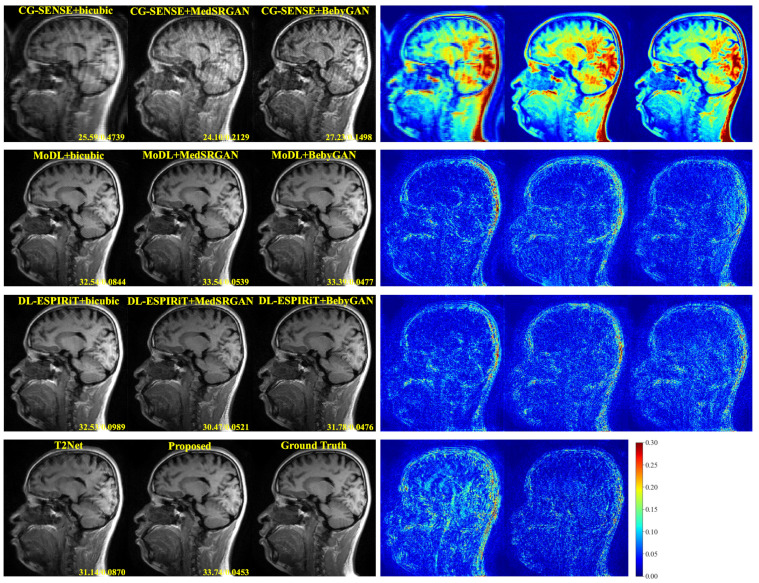
Visual comparison of each method for a slice of sagittal view in the brain dataset.

**Figure 7:**
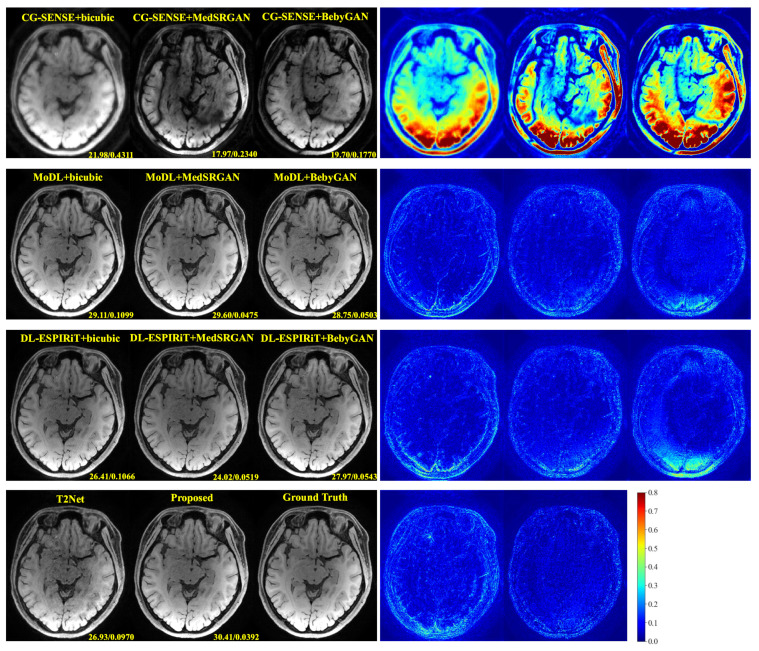
Visual comparison of each method for a brain image in the VWI dataset.

**Figure 8:**
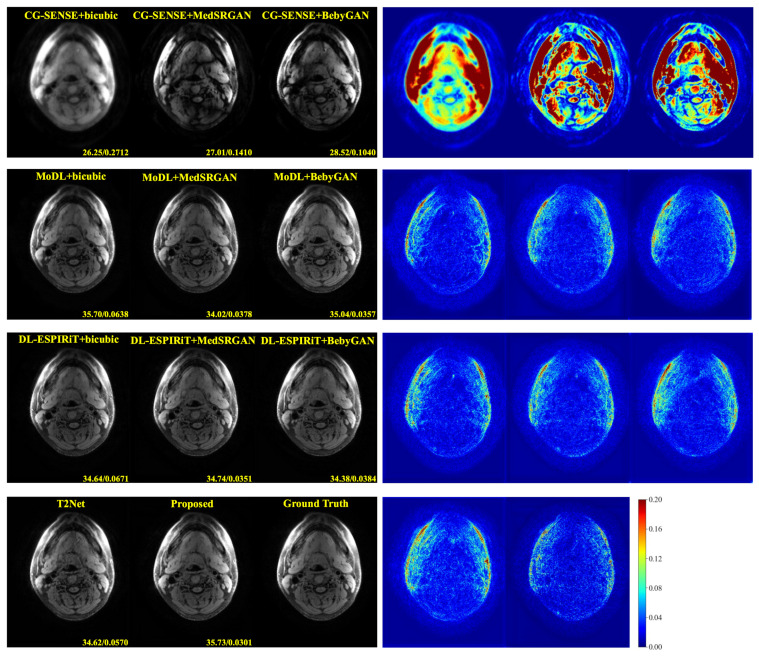
Visual comparison of each method for a neck image in the VWI dataset.

For a more intuitive comparison, we provide visual comparisons for each method. In Figure 5 and Figure 6, we illustrate the axial and sagittal views of the 2D brain dataset, respectively. In Figure 7 and Figure 8, we display the brain image and neck image of the 3D VWI dataset. For each method, we show the restorations along with the corresponding error maps. The images are placed on the left, the error maps on the right, and the PSNR and LPIPS metrics of each image are shown at the bottom right corner. From the error maps, it is evident that our proposed method consistently produces high-quality results with improved visual fidelity and better retention of structural information compared to the other methods.

**Figure 9:**
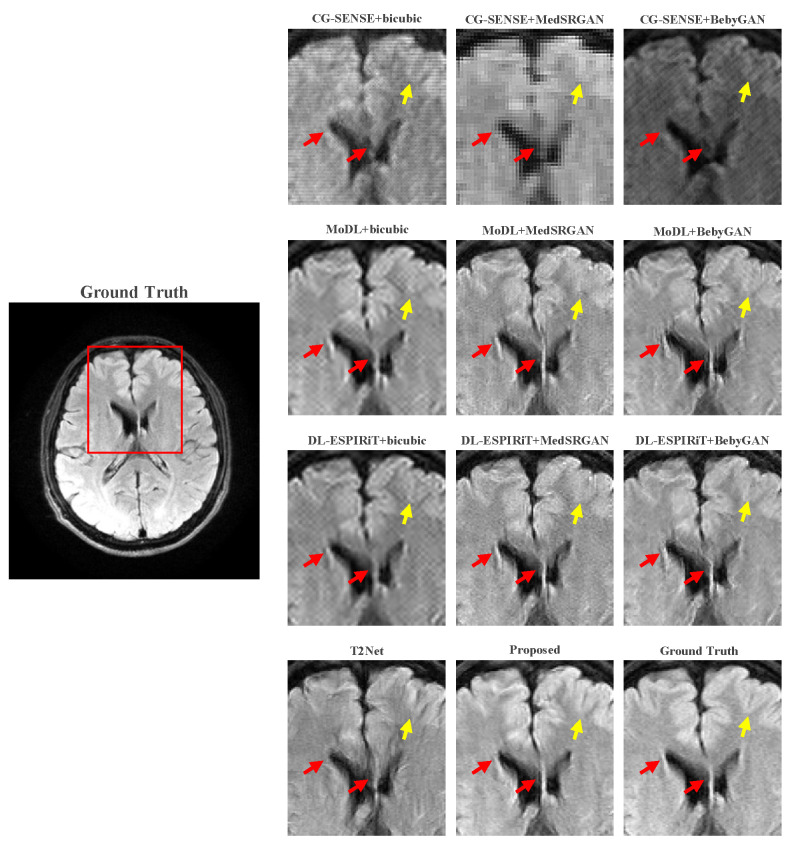
Zoomed-in view of the brain dataset comparison experiment.

**Figure 10:**
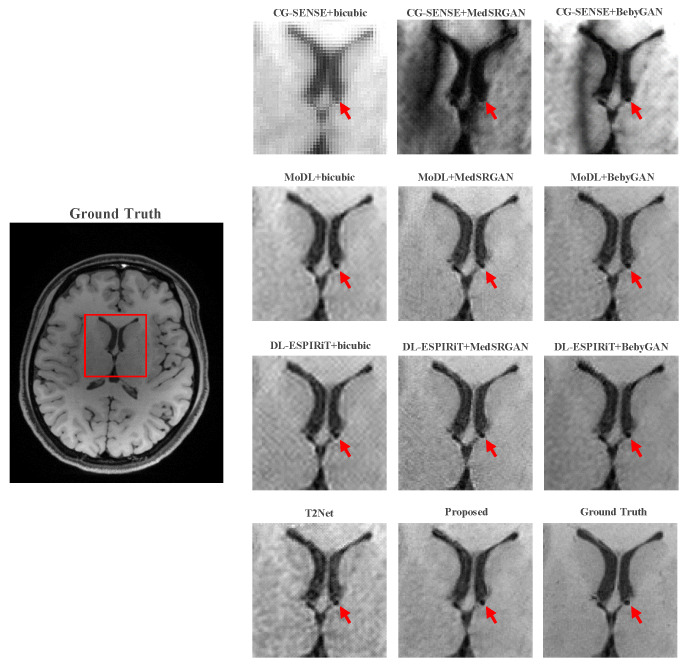
Zoomed-in view of the VWI dataset comparison experiment.

Figure 9 and Figure 10 present the zoomed-in images of the enclosed parts in the ground truth. Our proposed method can faithfully recover the fine structures, as indicated by the red arrows, and successfully preserve the image contrast, as pointed out by the yellow arrow. The high-fidelity restoration achieved by our method in these zoomed-in regions further demonstrates its ability to faithfully recover fine details and enhance image quality.

## 5. Discussion

Although our experimental results have demonstrated the validity of our proposed model, there are some limitations that should be noted. Firstly, the VWI dataset used in our study is a 3D dataset, while our model is designed as a 2D model, neglecting the correlation information between slices. Secondly, all the images used in our experiments are brain or neck images, and the performance of our model on images of other parts of the human body remains to be investigated. Finally, while our model has shown promising results in simulated scenarios, its performance in real medical settings needs to be further explored.

To address these limitations, we plan to develop better models that can leverage the correlation information between slices in 3D datasets and consider introducing data from other parts of the human body. Additionally, we aim to validate the effectiveness of our proposed methods in clinical settings by testing them on real undersampled LR data from a diverse range of participants. This will further validate the potential and utility of our method in medical imaging and contribute to more accurate and efficient MR image restoration for clinical diagnosis and research.

## 6. Conclusions

In this paper, we have introduced a novel multi-task framework for joint MR reconstruction and image super-resolution. Our proposed method incorporates two main modules: the Rec Module responsible for image reconstruction in the LR space and the SR Module which extracts features in the LR space and recovers the SR image. To enhance information exchange between the modules, we introduced the SFT layer to transmit features of the reconstructed image to the SR Module, facilitating super-resolution. The experimental results conducted on 2D brain data and 3D VWI data have demonstrated the superior performance of our proposed method in both quantitative and qualitative evaluations. The comparisons with other state-of-the-art methods have shown that our approach consistently generates high-quality images with enhanced visual fidelity and structural information.

## Figures and Tables

**Table 1:** Quantitative results of ablation study on the brain dataset. The average value and standard deviation are given for each metric. The figures in bold are the best results.

Metric	PSNR	SSIM	LPIPS
SR	28.88 ± 2.40	0.8462 ± 0.0446	0.0559 ± 0.0155
Recon	30.23 ± 2.43	0.8483 ± 0.0416	0.1429 ± 0.0178
Recon-SR	29.27 ± 2.45	0.8320 ± 0.0479	0.0537 ± 0.0146
Recon+SR	29.40 ± 2.62	0.8625 ± 0.0403	0.0482 ± 0.0117
Proposed	**30.32 ± 2.42**	**0.8682 ± 0.0418**	**0.0409 ± 0.0111**

**Table 2:** Quantitative results of ablation study on the VWI dataset. The average value and standard deviation are given for each metric. The figures in bold are the best results.

Metric	PSNR	SSIM	LPIPS
SR	31.93 ± 3.11	0.8657 ± 0.0324	0.0568 ± 0.0114
Recon	32.34 ± 4.60	0.8912 ± 0.0355	0.1089 ± 0.0306
Recon-SR	32.32 ± 3.22	0.8750 ± 0.0264	0.0513 ± 0.0102
Recon+SR	32.72 ± 3.00	0.8856 ± 0.0293	0.0425 ± 0.0074
Proposed	**32.91 ± 3.03**	**0.8948 ± 0.0288**	**0.0383 ± 0.0062**

**Table 3:** Quantitative results of different models on the brain dataset. The average value and standard deviation are given for each metric. The figures in bold are the best results. The average computation time in seconds for all models on the brain dataset is also provided.

Metric	PSNR	SSIM	LPIPS	Computation Time
CG-SENSE+bicubic	22.41 ± 1.90	0.5805 ± 0.0606	0.4425 ± 0.0533	0.0241
CG-SENSE+MedSRGAN	23.04 ± 2.01	0.6038 ± 0.0567	0.1812 ± 0.0396	0.0517
CG-SENSE+BebyGAN	23.33 ± 3.76	0.7151 ± 0.0593	0.1570 ± 0.0354	0.0647
MoDL+bicubic	29.33 ± 2.48	0.8463 ± 0.0397	0.0929 ± 0.0190	0.0659
MoDL+MedSRGAN	29.62 ± 2.47	0.8466 ± 0.0438	0.0467 ± 0.0121	0.0935
MoDL+BebyGAN	29.84 ± 2.64	0.8590 ± 0.0464	0.0476 ± 0.0129	0.1048
DL-ESPIRiT+bicubic	29.44 ± 2.88	0.8538 ± 0.0388	0.0996 ± 0.0240	0.0220
DL-ESPIRiT+MedSRGAN	29.43 ± 2.93	0.8319 ± 0.0488	0.0477 ± 0.0146	0.0486
DL-ESPIRiT+BebyGAN	29.69 ± 3.07	0.8596 ± 0.0455	0.0455 ± 0.0141	0.0591
T2Net	28.05 ± 2.39	0.8236 ± 0.0436	0.0685 ± 0.0174	0.1820
Proposed	**30.32 ± 2.42**	**0.8682 ± 0.0418**	**0.0409 ± 0.0111**	0.0775

**Table 4:** Quantitative results of different models on the VWI dataset. The average value and standard deviation are given for each metric. The figures in bold are the best results. The average computation time in seconds for all models on the VWI dataset is also provided.

Metric	PSNR	SSIM	LPIPS	Computation Time
CG-SENSE+bicubic	26.15 ± 2.95	0.7323 ± 0.1015	0.2952 ± 0.0769	0.0540
CG-SENSE+MedSRGAN	24.71 ± 4.40	0.7616 ± 0.0835	0.1799 ± 0.0416	0.1068
CG-SENSE+BebyGAN	25.31 ± 3.41	0.7886 ± 0.0681	0.1466 ± 0.0366	0.1236
MoDL+bicubic	32.24 ± 3.31	0.8753 ± 0.0284	0.0953 ± 0.0209	0.1375
MoDL+MedSRGAN	32.30 ± 3.03	0.8651 ± 0.0278	0.0448 ± 0.0088	0.1873
MoDL+BebyGAN	32.71 ± 3.46	0.8933 ± 0.0312	0.0481 ± 0.0103	0.2056
DL-ESPIRiT+bicubic	32.49 ± 3.49	0.8727 ± 0.0278	0.0859 ± 0.0192	0.0402
DL-ESPIRiT+MedSRGAN	31.82 ± 4.32	0.8707 ± 0.0307	0.0454 ± 0.0076	0.0902
DL-ESPIRiT+BebyGAN	32.43 ± 3.22	0.8707 ± 0.0260	0.0491 ± 0.0088	0.1073
T2Net	30.75 ± 3.59	0.8322 ± 0.0353	0.0808 ± 0.0191	0.3426
Proposed	**32.91 ± 3.03**	**0.8948 ± 0.0288**	**0.0383 ± 0.0062**	0.1385

## Data Availability

All of our data is unavailable due to privacy or ethical restrictions.
